# From H5N1 to HxNy: An epidemiologic overview of human infections with avian influenza in the Western Pacific Region, 2003–2017

**DOI:** 10.3565/wpsar.2018.9.2.001

**Published:** 2018-07-06

**Authors:** Sarah Hamid, Yuzo Arima, Erica Dueger, Frank Konings, Leila Bell, Chin-Kei Lee, Dapeng Luo, Satoko Otsu, Babatunde Olowokure, Ailan Li, WPRO Health Emergencies Programme Team

**Affiliations:** a WHO Regional Office for the Western Pacific.; b National Institute of Infectious Diseases, Japan.; c WHO Country Office China.; d WHO Country Office Lao People’s Democratic Republic.; e WHO Country Office Viet Nam.; f Influenza Division, Centers for Disease Control and Prevention, Atlanta, GA, USA.

Since the first confirmed human infection with avian influenza A(H5N1) virus was reported in Hong Kong Special Administrative Region SAR (China) in 1997, sporadic zoonotic avian influenza viruses causing human illness have been identified globally with the World Health Organization (WHO) Western Pacific Region as a hotspot. A resurgence of A(H5N1) occurred in humans and animals in November 2003. Between November 2003 and September 2017, WHO received reports of 1838 human infections with avian influenza viruses A(H5N1), A(H5N6), A(H6N1), A(H7N9), A(H9N2) and A(H10N8) in the Western Pacific Region. Most of the infections were with A(H7N9) (*n* = 1562, 85%) and A(H5N1) (*n* = 238, 13%) viruses, and most (*n* = 1583, 86%) were reported from December through April. In poultry and wild birds, A(H5N1) and A(H5N6) subtypes were the most widely distributed, with outbreaks reported from 10 and eight countries and areas, respectively.

Regional analyses of human infections with avian influenza subtypes revealed distinct epidemiologic patterns that varied across countries, age and time. Such epidemiologic patterns may not be apparent from aggregated global summaries or country reports; regional assessment can offer additional insight that can inform risk assessment and response efforts. As infected animals and contaminated environments are the primary source of human infections, regional analyses that bring together human and animal surveillance data are an important basis for exposure and transmission risk assessment and public health action. Combining sustained event-based surveillance with enhanced collaboration between public health, veterinary (domestic and wildlife) and environmental sectors will provide a basis to inform joint risk assessment and coordinated response activities.

Avian influenza viruses occur naturally among wild aquatic birds and cause occasional outbreaks in domestic poultry and other animal species. (*1*) They do not normally infect humans, though certain subtypes, such as avian influenza A(H5), A(H7) and A(H9) have caused sporadic human infections. Clinical outcomes range from mild illness to death. (*2*) Co-circulation of influenza A viruses in human and animal reservoirs in shared habitats provides opportunities for these viruses to reassort and acquire a genetic composition that could facilitate sustained human-to-human transmission with potential pandemic consequences. (*3*, *4*)

The pandemic potential of avian influenza viruses gained larger recognition in 1997 when the first known human infection with avian influenza A(H5N1) virus was reported in Hong Kong Special Administrative Region SAR (China). (*5*) During this event, 18 human infections, including six deaths, were reported. (*6*) Thereafter, the number of countries reporting human infections with A(H5N1) virus increased, especially between 2003 and 2008. As of September 2017, outbreaks associated with A(H5N1) viruses in domestic poultry and wild birds have occurred in more than 60 countries, and sporadic human infections with A(H5N1) viruses have been reported in 16 countries. A 53% case fatality has been reported among human cases of A(H5N1), which has been associated with severe pneumonia. (*7*) In addition to A(H5N1), other novel zoonotic influenza viruses infecting humans have emerged, including A(H5N6), A(H7N9), A(H10N8), A(H6N1) and a novel A(H1N2) variant. (*1*, *8*) The Western Pacific Region has reported more than one quarter (238/860) of global A(H5N1) cases and is the second most affected region. (*9*) Moreover, the recently identified zoonotic strains A(H7N9), A(H5N6), A(H6N1) and A(H10N8) emerged in the Western Pacific Region. (*10*)

Regional and international tools and frameworks have been implemented to address the threat of pandemic influenza and other emerging diseases. Regional and country-specific analyses are important as case fatality, demographic characteristics, seasonality and the clade or subclade of viruses have been observed to vary across regions. (*11*) In the Western Pacific Region, the *Asia Pacific Strategy for Emerging Diseases and Public Health Emergencies* (APSED III) is an action framework to strengthen public health sector capacity to manage and respond to emerging disease threats and to support progress towards implementation of the International Health Regulations (2005) (IHR). (*12*) APSED III promotes the sharing and use of information from multiple data sources for surveillance and risk assessment and aligns with global initiatives such as the One Health approach for multisectoral collaboration and communication in public health. (*13*) Member State notification to the World Health Organization (WHO) of zoonotic influenza virus infections in humans is mandated under the IHR, and WHO has maintained an epidemiologic database of human infections with zoonotic influenza viruses reported since 2003. Infections with highly pathogenic avian influenza A virus in birds and low pathogenic influenza H5 and H7 viruses in poultry are notifiable to the World Organization for Animal Health (OIE) under the Terrestrial Animal Health Code. (*14*) Data on animal outbreaks are available through OIE and the Food and Agriculture Organization of the United Nations (FAO) Global Animal Disease Information System (EMPRES-i). (*15*, *16*) EMPRES-i consolidates disease events worldwide using information from official and unofficial sources including reports by OIE chief veterinary officers. (*15*) The public availability of these data contributes to the compilation, analysis, interpretation and dissemination of information on avian influenza viruses in humans and animals.

In addition to these international frameworks, the WHO Global Influenza Surveillance and Response System (GISRS) is a laboratory network that collects data on influenza viruses circulating globally to inform vaccine composition recommendations, conduct risk assessments and monitor antiviral susceptibility. (*17*) In the Western Pacific Region, GISRS includes three WHO collaborating centres, six H5 reference laboratories and 21 national influenza centres (NICs) in 15 countries and areas. (*18*) WHO regularly produces global and regional updates on avian influenza virus activity and publishes timely information on novel human infections with zoonotic influenza viruses through *Disease Outbreak News*. (*7*, *19*-*21*)

While reports of human infections with A(H5N1) virus have declined since 2013, notifications of human infections with A(H7N9) and other avian influenza viruses have increased, highlighting the continued threat posed by these A(HxNy) viruses. Analyses of avian influenza virus infections in humans and outbreaks in birds can provide a basis for multisectoral risk assessments. This report summarizes the descriptive epidemiology of reported laboratory-confirmed human infection with avian influenza viruses in the Western Pacific Region along with reported outbreaks of these viruses in birds from the resurgence of A(H5N1) activity in November 2003 through the fifth epidemic of A(H7N9) ending on 30 September 2017.

## Methods

Data on human infections with avian influenza virus subtypes were summarized by person, place and time; bird infections were summarized by place and time. The starting date for this analysis was November 2003 when there was a resurgence in reported A(H5N1) activity in both humans and animals across several countries. (*22*)

Data on human infections with onset dates from November 2003 through September 2017 in the Western Pacific Region were based on official notifications to WHO under IHR. These notifications were primarily reported from National IHR Focal Points to the Western Pacific Regional IHR Contact Point. Notifications included the avian influenza virus subtype, demographic and epidemiologic information available at the time of reporting; information on virus clade was not included in reports. Infections notified and summarized in this analysis were with avian influenza subtypes A(H5N1), A(H5N6), A(H6N1), A(H7N9), A(H9N2) and A(H10N8). For A(H7N9), information regarding clusters of infection and virus pathogenicity in poultry was also included.

Data on infections with these influenza virus subtypes in birds in the Western Pacific Region were extracted from the EMPRES-i database, which includes reports of avian influenza events involving both low and highly pathogenic viruses—the former cause few or no clinical signs and the latter, severe clinical signs in poultry. The database was queried for confirmed events in domestic, wild and captive birds observed from January 2003 through September 2017. For low and highly pathogenic H5 and H7 viruses notifiable to OIE, (*14*) records reported by official sources including national authorities, OIE, FAO or laboratories were extracted. For non-H5 and non-H7 low pathogenic viruses not notifiable to OIE, such as A(H6N1), A(H9N2) and A(H10N8), outbreaks and detections reported in publications were also extracted from EMPRES-i. Data were summarized and analysed in SAS (University Edition, Cary, NC, USA) and Microsoft Excel and mapped in ArcGIS (Esri, Redlands, CA, USA) to describe the demographic, temporal and spatial characteristics of avian influenza virus activity in the Region.

## Results

**Figure 1:**
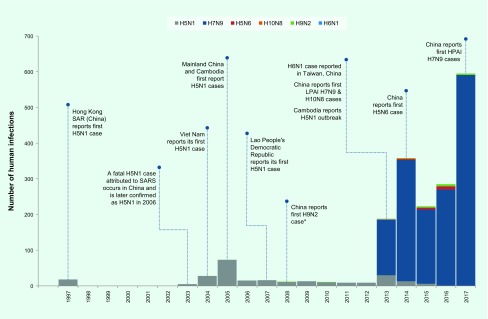
**Timeline of human infections with avian influenza virus subtypes in the Western Pacific Region, May 1997–September 2017**

**Figure 2:**
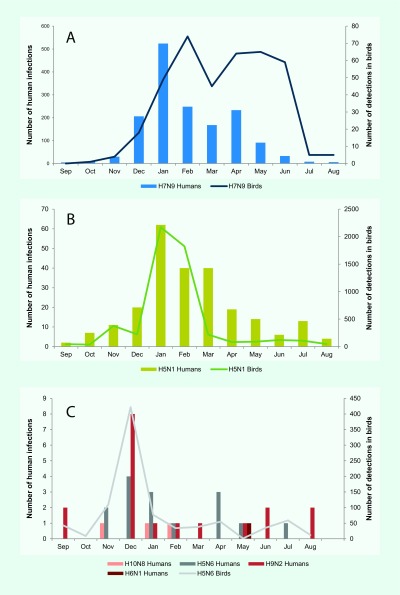
Reported human infections with avian influenza viruses and events in birds in the Western Pacific Region by month, November 2003–September 2017*

From November 2003 through September 2017, 1838 human infections with six avian influenza viruses in the Western Pacific Region were reported to WHO. The majority of infections were with A(H7N9) (*n* = 1562, 85%) and A(H5N1) (*n* = 238, 13%) viruses. Infections with A(H5N1) predominated until 2013 when reports of A(H7N9) emerged in China (**Fig. 1**). The majority (*n* = 1583, 86%) of human infections were reported from December through April. While this seasonality was largely driven by A(H7N9) and A(H5N1) cases, most A(H5N6) and A(H9N2) cases (*n* = 22, 65%) and all three A(H10N8) cases were also reported during this period (**Fig. 2**). With the exception of A(H5N1) and A(H6N1) viruses, all human infections in the Region were reported from, or associated with history of travel to, China.

[**insert** Figure 1]

[**insert** Figure 2]

**Figure 3:**
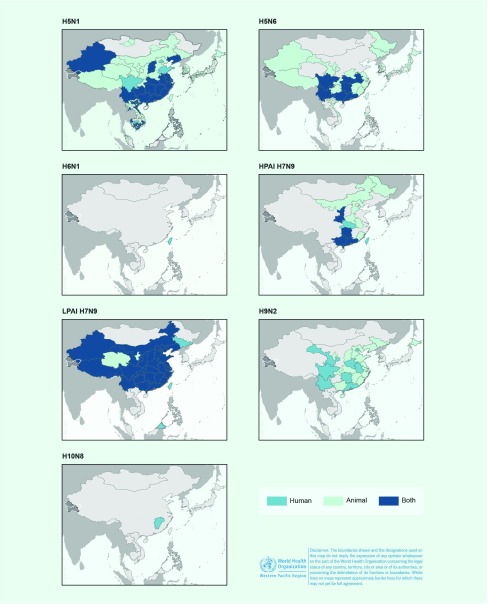
Map of avian influenza virus detections reported in human and birds in the Western Pacific Region, November 2003–September 2017*

In birds, A(H5N1) and A(H5N6) viruses were the most widely distributed in the Western Pacific Region, and outbreaks were reported from 10 and eight countries and areas, respectively (**Fig. 3**). Low pathogenic avian influenza (LPAI) A(H9N2) viruses have been detected in poultry populations of five Western Pacific Region countries and areas since 2004. As of 30 September 2017, poultry infections with A(H7N9) virus have not been reported in the Western Pacific Region outside of China.

[**insert** Figure 3]

### Human infections with avian influenza A(H5N1) viruses

From November 2003 through September 2017, 238 laboratory-confirmed human infections with avian influenza A(H5N1) were reported to WHO from four countries in the Western Pacific Region: Cambodia (*n* = 56), China (including Hong Kong Special Administrative Region SAR) (*n* = 53), the Lao People's Democratic Republic (*n* = 2) and Viet Nam (*n* = 127) (**Table 1**). The most recently reported A(H5N1) human infection in the Western Pacific Region had symptom onset in December 2015 and was from China. The overall case fatality rate (CFR) at the time of report was 56% (134/238) with 37 deaths in Cambodia (CFR 66%), 31 deaths in China (CFR 58%) and 64 deaths in Viet Nam (CFR 50%). Both cases in the Lao People's Democratic Republic were reported as fatal. Seasonally, the majority of cases (*n* = 142, 60%) occurred from January through March (**Fig. 2**). Reports of A(H5N1) infections in humans peaked from November 2003 through December 2005 (*n* = 106) when notifications from Viet Nam (*n* = 93) surged and later from January 2013 through March 2014 when there was an outbreak in Cambodia (*n* = 35) (**Fig. 1**).

**Figure 4:**
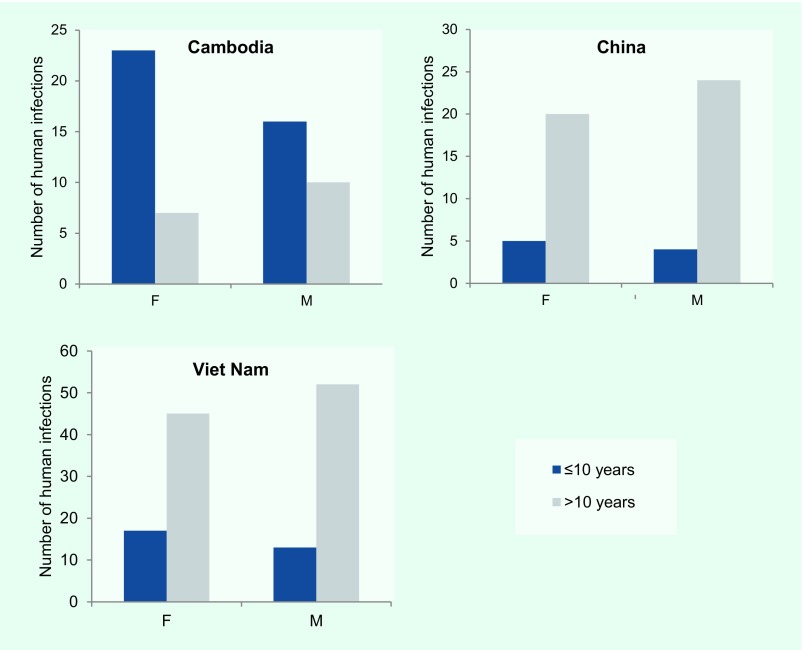
**Reported cases of human infections with avian influenza A(H5N1) virus in Cambodia, China and Viet Nam by age and sex, November 2003–September 2017**

[**insert** Figure 4]

**Table 1:** Demographic, geographic and temporal characteristics of avian influenza virus subtypes reported in the Western Pacific Region, November 2003–September 2017

-	Influenza A virus subtype					
-	H5N1	H7N9	H5N6	H9N2	H10N8	H6N1
Human infections, *n*	238	1562	16	18	3	1
Median age (range), years	20 (< 1–81)	57 (< 1–91)	40 (11–65)	3 (< 1–86)	73 (55–75)	20
Male	23 (< 1- 81)	57 (1–91)	44 (25–58)	2 (< 1–86)	75	–
Female	18 (< 1–75)	56 (< 1–85)	37 (11–65)	4 (< 1–57)	55, 73	20
Geographic spread*						
Countries/areas affected (humans)	Cambodia, China, Lao People's Democratic Republic, Viet Nam	China, Malaysia (travel history to mainland China)	China	China (including Hong Kong Special Administrative Region SAR with travel history to mainland China)	China	China, Taiwan, China
Countries/areas affected (birds)	Cambodia, China (including Hong Kong Special Administrative Region SAR and China, Taiwan, China), Japan, Lao People's Democratic Republic, Malaysia, Mongolia, Republic of Korea, Viet Nam	China (including Hong Kong Special Administrative Region SAR, Macao SAR and China, Taiwan, China)	China (including Hong Kong Special Administrative Region SAR and China, Taiwan, China), Japan, Lao People's Democratic Republic, Philippines, Republic of Korea, and Viet Nam	China (including Hong Kong Special Administrative Region SAR), Japan, Republic of Korea and Viet Nam	No reports in EMPRES-i	No reports in EMPRES-i

Across the Region, 50% (*n* = 119) of A(H5N1) cases were female; the sex distribution was similar when stratified by country, with females comprising 49% (*n* = 62) of cases in Viet Nam, 47% (*n* = 25) in China and 54% (*n* = 30) in Cambodia. In the Lao People's Democratic Republic both cases were female. The overall median age of cases was 20 years (range: < 1–81 years), but age distributions differed by country (**Fig. 4**). The median age of cases in Cambodia (6 years, range: < 1–58 years) was considerably lower than that observed in China (27 years, range: 2–75 years), Viet Nam (23 years, range: < 1–81 years) and the Lao People's Democratic Republic (15 and 42 years). These differences in age distributions remained when stratified by sex, with a predominance of paediatric cases in Cambodia regardless of sex (**Fig. 4**). For all countries, however, female cases tended to be younger than male cases (**Fig. 4**). Data on poultry exposure were available for 152 of 238 (64%) cases; of these cases, 95% (*n* = 145) reported contact with poultry.

### Avian influenza A(H5N1) virus in birds

Since late 2003, high mortality associated with A(H5N1) virus has been observed in poultry and wild birds in the Western Pacific Region. All reported viruses were highly pathogenic. Events (*n* = 5344) were reported from 10 countries and areas (**Table 1**, **Fig. 3**). The majority (*n* = 4037, 76%) were reported in Viet Nam during 2004 and 2005. The reported number of events in avian populations decreased steadily from 2004 to 2006, rose slightly in 2007 and has since declined. In March 2017, however, Malaysia reported its first A(H5N1) poultry outbreak since 2006. Events were reported year-round but most frequently (*n* = 4597, 86%) from November through February, coinciding with the months when A(H5N1) infections in humans were most frequently reported (**Fig. 2**).

### Human infections with avian influenza A(H5N6) viruses

As of 30 September 2017, 16 laboratory-confirmed human infections with avian influenza A(H5N6) virus had been reported to WHO from China. At the time the cases were reported, four (25%) cases had died. The first human case was reported in May 2014 in Sichuan province and was associated with infected poultry. (*23*) Subsequent infections were detected between December 2014 and November 2016 from the eastern province of Anhui (*n* = 1), the southern provinces of Hunan (*n* = 3), Guangdong (*n* = 7), Guangxi (*n* = 1), Yunnan (*n* = 2) and the central province of Hubei (*n* = 1) (**Fig. 3**).

Ages ranged from 11 to 65 years (median 40 years). Males (7 of 16 cases) were older compared to females (**Table 1**). Contact with poultry or wild birds was reported in all 13 cases for whom exposure history was known.

### Avian influenza A(H5N6) virus in birds

The first outbreaks of A(H5N6) virus in poultry were reported in March 2014 in Xayabury, Lao People's Democratic Republic and in Sichuan, China in May 2014. However, the virus had been isolated in December 2013 from an environmental sample collected in a live poultry market in Jiangsu Province. (*24*) Since then, the geographic distribution of reported events gradually expanded, affecting eight countries and areas by September 2017 (**Table 1**, **Fig. 3**). All events involved highly pathogenic avian influenza (HPAI) A(H5N6), except for two events involving LPAI A(H5N6) in Hunan Province, China and Louangphabang, Lao People's Democratic Republic. A(H5N6) events in birds were widespread in most of the affected countries (**Fig. 3**).

The majority of events were reported from the Republic of Korea (*n* = 386, 43%) followed by mainland China (*n* = 260, 9%). Across the Region, events were reported year-round with some variation in circulation among countries. In mainland China, Japan and the Republic of Korea, the largest number (*n* = 417, 46%) of events occurred in December. The Lao People's Democratic Republic reported three events in March, July and October. In Viet Nam events were reported every month of the year with no clear seasonality. The Philippines reported its first A(H5N6) outbreaks in July and August 2017.

### Human infections with avian influenza A(H7N9) viruses

**Figure 5:**
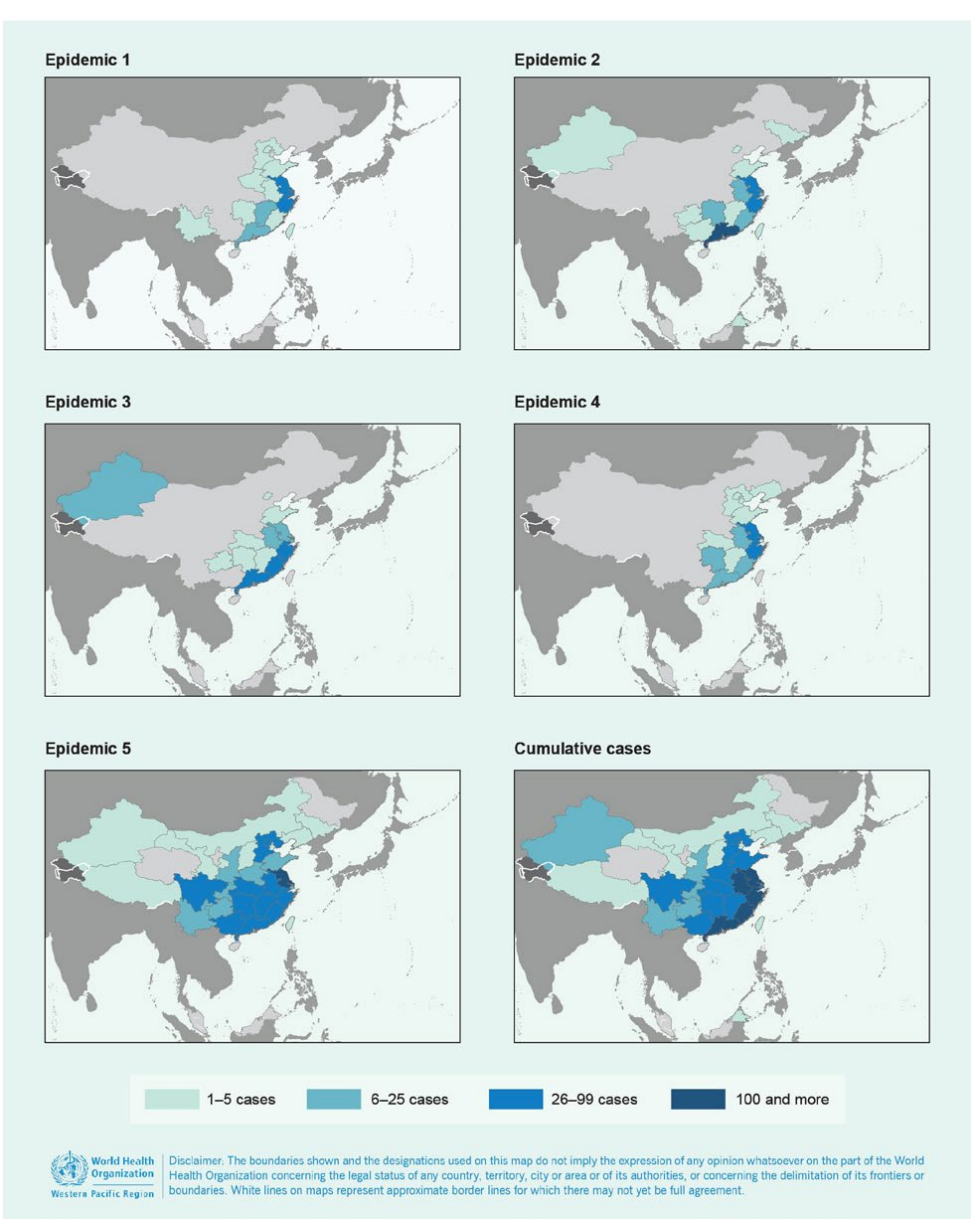
Geographic distribution of reported cases of human infections with avian influenza A(H7N9) virus in the Western Pacific Region, March 2013–September 2017*

Between 31 March 2013 and 30 September 2017, 1564 laboratory-confirmed human infections with avian influenza A(H7N9) virus were reported to WHO, occurring in five annual epidemics (defined as reported case onset from 1 October to 30 September of the following year). The outbreak began in China in March 2013 with two patients from Shanghai and one from Anhui. The geographic distribution of reported cases has shifted and expanded over time with cases reported from 27 mainland China provinces and municipalities, several of which are along international borders, as well as from Hong Kong Special Administrative Region SAR, Macao SAR and China, Taiwan, China (**Fig. 5**). In addition, cases associated with travel to China were reported in Malaysia (*n* = 1) and Canada (*n* = 2). (*7*)

[**insert** Figure 5]

The majority (*n* = 1381, 88%) of cases occurred from December to April each year with a few sporadic cases occurring during the summer months (**Fig. 2**). The peak of A(H7N9) infections was in January, with the exception of 2013 when notifications peaked in April. The majority of cases were reported from Zhejiang (*n* = 310, 20%), Guangdong (*n* = 258, 16%) and Jiangsu (*n* = 252, 16%) provinces on China’s eastern coast (**Fig. 5**).

The median age of cases was 57 years (range: < 1–91 years), and 67% (*n* = 1054) of cases were aged 50 years and older. Overall, approximately 70% of A(H7N9) cases were male (**Table 2**), but the proportion differed by age; among those aged 0–24 years, males comprised 49% (*n* = 38) of cases, but among those 25 years of age and older, 67% (*n* = 1055) were male. Among the latter, further age group stratification (25–34, 35–44, 45–54, 55–64, 65+ years) indicated that the predominance of males was similar across these age groups (range: 68% to 72%).

**Table 2:** Characteristics of A(H7N9) epidemics, March 2013–September 2017

-	Epidemic (year)*				
-	2013	2013–2014	2014–2015	2015–2016	2016–2017
**Human infections,** *n*	**135**	**320**	**224**	**119**	**766**
**Male,** *n* **(%)**	**97 (72)**	**218 (68)**	**154 (69)**	**78 (66)**	**546 (71)**
**Median age (range), years**	**61 (2–91)**	**57 (< 1–88)**	**56 (1–88)**	**58 (13–91)**	**57 (3–91)**
**Clusters,** *n*	**4**	**9**	**6**	**6**	**14**
**Month of peak notifications**	**April**	**January**	**January**	**January**	**January**
Provinces** reporting human infections, *n*	**13**	**17**	**15**	**15**	**30**
Provinces** reporting detections in birds, *n*	**11**	**12**	**14**	**10**	**27**

* Epidemics are defined as 1 October to 30 September of the following year with the exception of the first epidemic that started in April 2013.

** Within China, provinces refer to China’s provincial administrative units, which include province, autonomous region, municipality and special administrative region. Information on detections in birds is based on data in the EMPRES-i system as of November 2017.

The sex and age distribution of cases were similar across epidemics with infections in men reported more frequently than in women (**Table 2**). However, shifts in the frequency as well as temporal and geographic distribution of cases were observed (**Table 2**, **Fig. 5**). The second epidemic year (1 October 2013–30 September 2014) was considerably higher in amplitude compared to the first and peaked in January rather than April. During the third (1 October 2014–30 September 2015) and fourth (1 October 2015–30 September 2016) epidemic years, the number of human infections reported declined, but there was no major change in the temporal distribution of cases compared to the second epidemic year (**Table 2**).

The fifth epidemic year of A(H7N9) activity in humans saw an epidemic that surpassed all previous years in amplitude and number of cases reported (*n* = 766), with peak activity observed in January 2017 consistent with trends observed in the second to fourth epidemic years. However, the increase in notifications started earlier than in previous years and expanded to the north and west with Jiangsu reporting the greatest number of cases (*n* = 148, 19%) and nine administrative regions (Chongqing, Gansu, Inner Mongolia, Shaanxi, Shanxi, Sichuan, Tibet, Yunnan provinces and Macao SAR) reporting cases for the first time.

As of 30 September 2017, WHO received reports of 39 clusters, three of which involved multiple provinces: two from Beijing and Hebei and one from Fujian and Zhejiang. Most were two-person clusters (*n* = 35, 90%), but three-person clusters also occurred (*n* = 4, 10%). With the exception of four clusters in health-care settings, all clusters involved household or family contacts. Clusters often involved cases that had exposure to live poultry or their environments; thus, it was not always possible to determine whether human-to-human transmission or common poultry exposure was the source of infection. Clusters increased in number but not in the size in the fifth epidemic (**Table 2**) with no apparent change in human-to-human transmission risk. (*25*)

While IHR notifications do not typically include virus pathogenicity, on 18 February 2017, the National Health and Family Planning Commission of China notified WHO of two previously reported human infections with viral sequences with changes at the haemagglutinin gene cleavage site that are associated with a transition from low to high pathogenicity in poultry. Since this announcement, 28 human cases have been identified with HPAI A(H7N9) from Guangdong, Guangxi, Hunan, Shaanxi and Hebei provinces, and China, Taiwan, China. Viral sequencing from one person in a family cluster of two sisters in Guangdong during the fifth epidemic was found to have these HPAI genetic markers. However, no viral samples from the other sister were available to determine if these markers were present in both cases.

### Avian influenza A(H7N9) virus in birds

Poultry surveillance for LPAI A(H7N9) has relied on targeted sampling because, by definition, infected poultry show little to no clinical signs of infection. In 2017, HPAI A(H7N9) was reported for the first time through active surveillance in a live bird market in Guangdong province and on a layer farm in Hunan province. Subsequent outbreaks were reported in nine other provinces in China. While the majority of A(H7N9) detections are LPAI viruses, recent viral changes found in human, poultry and environmental samples are associated with high pathogenicity in poultry. (*26*) Since it was first detected in 2013, low and/or highly pathogenic A(H7N9) viruses have been detected in poultry in 31 administrative areas of China, including Hong Kong Special Administrative Region SAR and Macao SAR. The number of provinces reporting virus detections has gradually increased over time (**Table 2**). However, some provinces that reported A(H7N9) detections in earlier years did not report infections in subsequent years.

Detections of LPAI A(H7N9) have been most frequent in the southern and eastern provinces, but reports have stemmed from 26 mainland administrative areas, from the northern province of Liaoning to the southern province of Hainan and the western provinces of Qinghai, Ningxia and Sichuan (**Fig. 3**). As of September 2017, the strain of A(H7N9) virus circulating in China has not been detected in poultry in other countries. Virus detections were most frequently reported between January and June.

### Other avian influenza A virus subtypes infecting humans and poultry

Other avian influenza viruses infecting humans in the Western Pacific Region include A(H9N2), A(H10N8) and A(H6N1).

Between December 2008 and September 2017, 18 human infections with avian influenza A(H9N2) virus were officially notified to WHO from China. Cases were reported from nine administrative areas: Hong Kong Special Administrative Region SAR (*n* = 3; all with travel history to Guangdong Province), Anhui (*n* = 1), Beijing (*n* = 1), Gansu (*n* = 1), Guangdong (*n* = 4), Henan (*n* = 1), Hunan (*n* = 5), Sichuan (*n* = 1) and Yunnan (*n* = 1) provinces. Cases had a median age of 33 years (range: < 1–86 years) and seven (39%) were male. At the time of notification, nine (50%) patients had been hospitalized and three manifested serious illness; none was fatal at the time of reporting.

LPAI A(H9N2) viruses circulate endemically among poultry in Asia. Since 2004, they have been detected in China (including Hong Kong Special Administrative Region SAR), Japan, the Republic of Korea and Viet Nam. A(H9N2) infections in poultry have been widespread in China (the EMPRES-i database includes international reference laboratory reports of detections from 23 of 34 administrative units from 2010 through 2014), but they have been found predominantly in eastern provinces. As of September 2017, no avian events had been reported in the Western Pacific Region since 2014.

Avian influenza A(H10N8) was responsible for three human infections in the Region as of March 2017. The first human infection was reported in a 73-year-old female in Jiangxi, China in December 2013; it was followed by two cases in the same province: a 55-year-old woman in January and a 75-year-old man in February 2014. All cases had poultry exposure and required hospitalization.

As a low pathogenic virus in birds, A(H10N8) is not notifiable to OIE and no events were recorded in the EMPRES-i system. However, isolation of A(H10N8) viruses from poultry and environmental samples, including in Jiangxi Province following detections in humans, has been reported in the scientific literature. (*27*-*29*)

In June 2013, a case of human A(H6N1) infection was reported to WHO from China, Taiwan, China. This was the first reported human infection with the virus. The case was a 20-year-old female hospitalized with mild pneumonia in May 2013. She had no known exposure to poultry and fully recovered.

Avian influenza A(H6N1) is an LPAI virus in birds and commonly circulates in the domestic bird population. (*30*-*32*) It is not a notifiable disease in animals, and no events were recorded in the EMPRES-i system.

## Discussion

Our regional analysis of human infection with avian influenza viruses reported from November 2003 through September 2017 revealed common patterns as well as variations in epidemiology across countries, age and time that may not be apparent from pooled global summaries or isolated country reports. In addition, assessing surveillance data from both the human and animal sectors provided a more complete overview of zoonotic influenza virus activity that can inform regional risk assessment and response efforts.

### Temporal trends

During the analysis period, notifications of human A(H5N1) infections followed similar temporal trends to those of A(H5N1) poultry outbreaks with initial increases in reports followed by declines by 2005. Reports of human A(H5N1) infections have remained low despite enhanced surveillance, awareness and reporting following the detection of other avian influenza virus subtypes. Declines in reported human and poultry infections despite enhanced surveillance activities indicate that a surveillance or reporting artefact is unlikely to explain the observed decline in A(H5N1).

While the incidences of human and animal A(H5N1) infections have likely declined, A(H7N9) has emerged as a new threat. The fifth A(H7N9) epidemic had the largest number of reported human infections to date with an earlier start and longer period of activity than previous seasons. (*33*) Human A(H7N9) infections occurred seasonally, coinciding with peak influenza detection in poultry as observed with A(H5N1) in other regions, (*4*, *14*) and similar to A(H5N1), A(H5N6), A(H9N2) and A(H10N8) in the Western Pacific Region. While the temporal correlation between human infections and poultry events may be due to increased influenza virus activity in birds that increases transmission potential to humans, surveillance bias could play a role (i.e. if surveillance is enhanced in humans once a poultry outbreak occurs or vice versa). Given the predominantly low pathogenic nature of A(H7N9) and the systematic targeted poultry sampling, this bias is unlikely. Enhanced surveillance and control measures at live bird markets, particularly during the cooler, drier months, could potentially reduce the risk of coinfection and reassortment.

HPAI A(H7N9) viruses have been detected recently; preliminary analyses based on eight cases indicated similar epidemiologic characteristics among humans infected with both low and highly pathogenic A(H7N9). (*34*) Animal studies have shown that HPAI A(H7N9) viruses can be transmitted through respiratory droplets, (*35*) but additional research is needed to determine the likelihood of this mode of transmission in humans.

### Geographic trends

Despite the extensive regional distribution of both A(H5N1) and A(H5N6) events in birds, only A(H5N1) has been reported in humans outside of China, excluding travel-associated A(H7N9) cases. (*36*) The absence of reported human or animal A(H7N9) infections in neighbouring countries that trade live poultry with China suggests that the A(H7N9) virus is currently limited to China. Based on live-bird trade patterns, the likelihood of A(H7N9) virus entry is considered moderate for the Lao People's Democratic Republic and Viet Nam and negligible for Cambodia, which has no known live poultry trade with China. (*37*) Nevertheless, the spatial distribution of reported A(H7N9) cases within China across epidemics and the presence of provinces in which only human cases have been detected may suggest undetected poultry infections.

### Demographic characteristics

Demographic characteristics of reported human infections varied. Aggregated age and sex distributions of human infections with A(H5N1) viruses in the Western Pacific Region were similar to global averages, (*7*) but epidemiologic patterns differed among countries. Accounting for sex, younger age groups were reported in Cambodia compared to China, the Lao People's Democratic Republic and Viet Nam; these differences were too large to be explained solely by differences in population age distributions. In addition, age distributions differed by sex; male cases tended to be older than their female counterparts. Such differences could arise from differential poultry exposure, health-care-seeking behaviour, case ascertainment or illness severity.

For non-A(H5N1) avian influenza virus infections in humans reported from China, the age and sex distributions also varied. Relative to A(H5N1) cases, A(H7N9) cases tended to be skewed towards older males, and, although numbers were small, A(H5N6) cases also tended to be older while A(H9N2) cases tended to be younger with more females. Explanations proposed for the difference in age and sex distribution of human A(H5N1) and A(H7N9) in China include differences in exposure patterns, increased susceptibility to serious disease after infection with A(H7N9) and case ascertainment bias. (*38*-*40*) Serological and epidemiologic data indicate that A(H5N1) infections may be more severe than A(H7N9) infections; A(H7N9) illness severity increases with patient age, and mild A(H7N9) infections in younger people may be underascertained. (*41*-*43*) While further studies are required to understand the factors and exposure patterns driving the epidemiology and to inform targeted prevention activities, basic surveillance data and descriptive epidemiology will continue to inform operational research and response. Population-level observations, particularly those related to poultry rearing and purchasing practices, will help generate preliminary hypotheses regarding risk factors for infection.

### Limitations

Interpretations based on surveillance data represent an important limitation in our assessments. While H5 and H7 serosurveys suggest limited asymptomatic illness, (*41*, *44*-*46*) even a low seroprevalence may indicate a substantial number of undetected cases and underestimations of the true burden and spectrum of zoonotic influenza infections. Surveillance and laboratory capacities vary among countries and between human and animal sectors; thus, country-level comparisons require caution. Moreover, within each country, the capacity to detect influenza viruses has evolved through national and partner support for influenza surveillance strengthening. (*47*) Surveillance biases may have affected the observed geographic distribution of cases as previously affected areas may have more complete surveillance and reporting. For example, regional variations in China in surveillance for pneumonia of unknown etiology led to increased surveillance in areas in which A(H7N9) cases had been detected relative to areas in which they had not. (*48*) While recognizing the role of possible ascertainment bias, surveillance and reporting enhancements have led to a more comprehensive understanding of the epidemiology of various influenza viruses circulating in the Region.

Event notification through IHR facilitates timely information sharing, greater understanding of an event as it unfolds and collaborative risk assessment to reduce the potential for international disease spread. Similarly, reporting to OIE is designed to facilitate information sharing and early warning and response to transboundary animal diseases. Thus, official IHR/OIE notifications include information available at the time of reporting that may not include sufficient exposure or outcome history to allow for an in-depth assessment.

Another limitation is potential missing data. Reports to WHO or OIE might not include all cases of detected human infections and poultry outbreaks. IHR (2005), which mandates the reporting of human infections with novel influenza viruses, did not come into effect until 2007. Thus, cases occurring before 2007 may not have been officially reported to WHO. (*49*-*54*) There are also human infections with avian influenza viruses after 2007 that are reported in the literature that have not been confirmed by national authorities or officially reported under the IHR. (*55*, *56*)

Duplicate event reports in the EMPRES-i database are another possibility. We did not include EMPRES-i records from publications for H5 and H7 viruses, which are notifiable to OIE, to avoid possible duplication of events officially reported by OIE, FAO or national authorities. As a result, events involving notifiable subtypes reported in scientific publications but not through official reports are not included in our summary.

## Conclusions

Despite these limitations, disseminating regional analyses can improve Member States’ situational awareness, knowledge of the epidemiology in neighbouring countries as well as of the broader regional perspective, and risk assessment and response efforts. This analysis specifically demonstrates the usefulness of combining multiple sources of surveillance data for better informed risk assessments, including those based on the WHO Tool for Influenza Pandemic Risk Assessment. (*57*) Moreover, using multiple sources of information helps to assess potential surveillance biases, thereby improving decision-making.

Further sporadic human infections with avian influenza viruses are likely to occur. Although A(H5N1) incidence may have declined, A(H7N9) virus has emerged, and other avian influenza viruses have been detected in recent years. In China, country of the origin of recently identified avian influenza virus strains, the poultry industry has expanded greatly in the past two decades. (*58*) In many areas, the close proximity of humans and animals increases the risk of human exposure to zoonotic influenza viruses. (*3*) As infected animals or contaminated environments are the primary sources for human infection, risk assessments should incorporate a One Health approach and gather information from all relevant sectors. Continued surveillance at the human–animal interface is imperative for all avian influenza viruses. Every sporadic human infection provides a virus with an opportunity to change its genetic makeup, increasing the possibility of an influenza virus with pandemic potential to arise. Strengthened communication and collaboration between animal and human health sectors at subnational, national, regional and global levels are necessary to monitor the dynamics of influenza virus activity. An APSED approach that aligns with One Health initiatives combining sustained event-based surveillance with enhanced collaboration between the human, animal (domestic and wildlife) and environmental sectors will provide a basis to inform joint risk assessment and coordinate response capacities.
